# Supplementary material for: Synapse specific alterations of autophagy are a hallmark of Danon disease
Source: bioRxiv. 2026 Apr 15:2026.04.14.718098. Preprint. [Version 1] doi: 10.64898/2026.04.14.718098 (PMC13105055; doi:10.64898/2026.04.14.718098)
Supplement: Supplement 1 [file media-1.docx]

**Synapse-specific alterations of autophagy are a hallmark of Danon disease**

Beatrice Terni^1,2^, Maria Quiles-Pastor^1,2^, Zoë Reynolds^3^, Kelsey Coppenrath^3^, Nikko-Ideen Shaidani^3^, Pablo Martínez San Segundo^1,2^, Shawn Adam^4^, Nicolás Riffo-Lepe^5,7^, Zachary Smith^6,7^, Marko Horb^3^, Carlos Aizenman^4,*^ and Artur Llobet^1,2,*^.

^1^ Laboratory of Neurobiology, Department of Pathology and Experimental Therapy, Institute of Neurosciences, University of Barcelona, 08907 L’Hospitalet de Llobregat, Barcelona, Spain

^2^ Bellvitge Biomedical Research Institute (IDIBELL), 08907 L’Hospitalet de Llobregat, Barcelona, Spain

^3^ Eugene Bell Center for Regenerative Biology and Tissue Engineering and National Xenopus Resource, Marine Biological Laboratory, Woods Hole, MA, USA

^4^ Department of Neuroscience, Brown University, Providence, RI, USA

^5^ Universidad de Concepción, Chile

^6^ University of Maryland Baltimore County, Baltimore MD

^7^  Neurobiology Course, Marine Biological Laboratory, Woods Hole, MA, USA

**SUPPLEMENTARY INFORMATION**

**
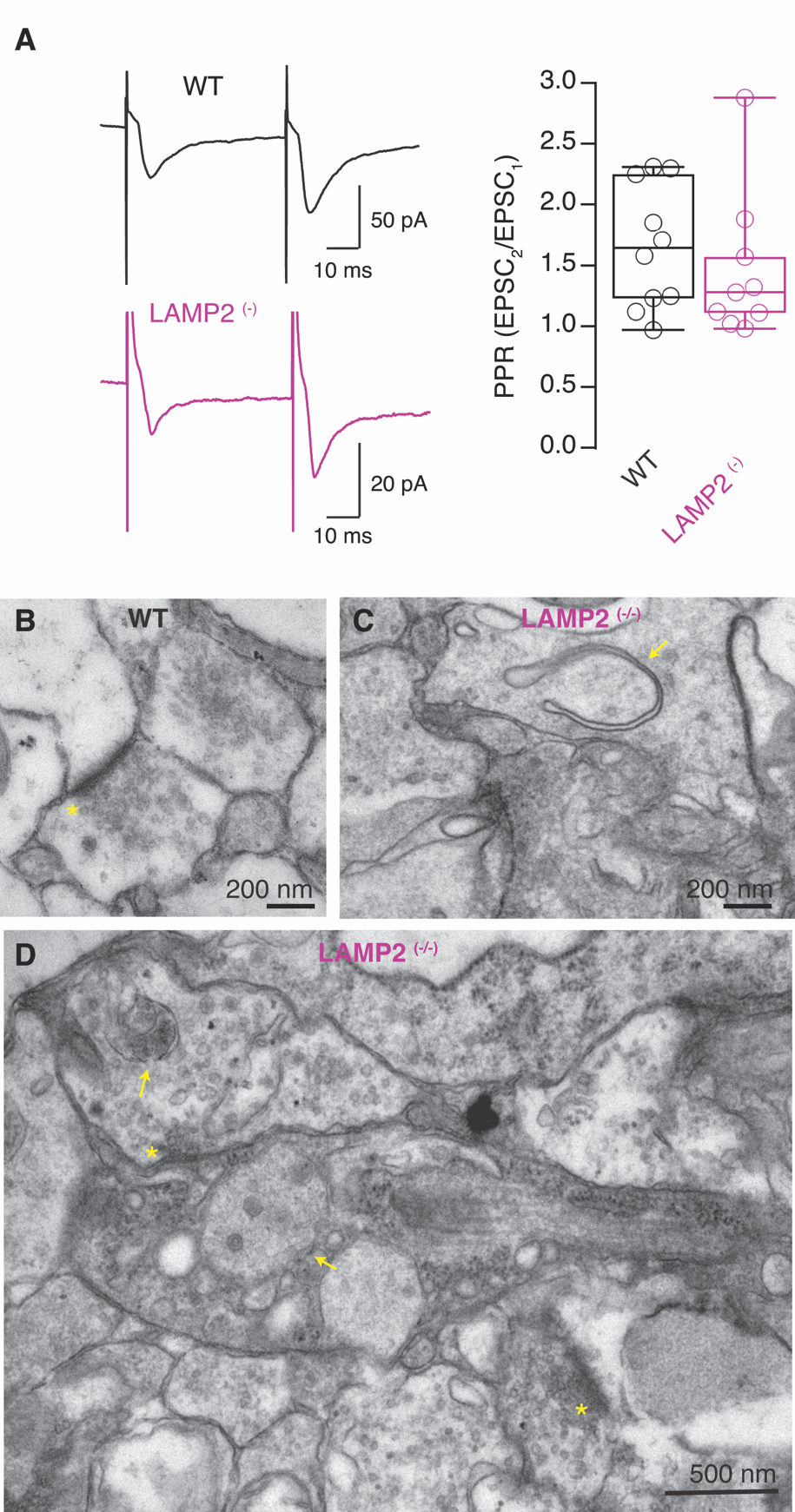
**

**Supplementary Figure 1. Centrally-evoked retinotectal activity remains normal in *LAMP2* ^(-)^ mutants. A)** Sample paired EPSCs recorded in tectal neurons, evoked by direct optic nerve stimulation, which bypasses the retina. Paired pulse facilitation, a measure of synaptic release competency remains unaltered in LAMP2 mutants, compared to WT tadpoles. **B)** Image of a conventional tectal synapse from a WT tadpole. **C,D)** Synapses found in the tectum of LAMP2 (-/-) tadpoles. Phagophores (C, arrow), were commonly observed. Autophagosomes were also obvious in pre and postsynaptic terminals (D, arrows). Asterisks indicate presynaptic active zones.
